# LMO1 functions as an oncogene by regulating TTK expression and correlates with neuroendocrine differentiation of lung cancer

**DOI:** 10.18632/oncotarget.25642

**Published:** 2018-07-03

**Authors:** Liqin Du, Zhenze Zhao, Milind Suraokar, Spencer S. Shelton, Xiuye Ma, Tzu-Hung Hsiao, John D. Minna, Ignacio Wistuba, Alexander Pertsemlidis

**Affiliations:** ^1^ Department of Chemistry and Biochemistry, Texas State University, San Marcos, Texas, USA; ^2^ Departments of Pathology and Thoracic/Head and Neck Medical Oncology, UT MD Anderson Cancer Center, Houston, Texas, USA; ^3^ Greehey Children’s Cancer Research Institute, UT Health Science Center at San Antonio, San Antonio, Texas, USA; ^4^ Department of Medical Research, Taichung Veterans General Hospital, Taichung, Taiwan; ^5^ Hamon Center for Therapeutic Oncology Research and Departments of Pharmacology and Internal Medicine, UT Southwestern Medical Center at Dallas, Dallas, Texas, USA; ^6^ Department of Translational Molecular Pathology, UT MD Anderson Cancer Center, Houston, Texas, USA; ^7^ Departments of Pediatrics and Cell Systems & Anatomy, UT Health Science Center at San Antonio, San Antonio, Texas, USA

**Keywords:** LMO1, lung cancer, neuroendocrine, TTK

## Abstract

LMO1 encodes a protein containing a cysteine-rich LIM domain involved in protein–protein interactions. Recent studies have shown that LMO1 functions as an oncogene in several cancer types, including non-small cell lung cancer (NSCLC). However, the function of LMO1 in other histological subtypes of lung cancer, such as small cell lung cancer (SCLC), was not investigated. In analyzing the expression of LMO1 across a panel of lung cell lines, we found that LMO1 expression levels were significantly and dramatically higher in SCLC cells, an aggressive neuroendocrine subtype of lung cancer, relative to NSCLC and normal lung cells. In NSCLC cells, LMO1 mRNA levels were significantly correlated with expression of neuroendocrine differentiation markers. Our *in vitro* investigations indicated that LMO1 had the general property of promoting cell proliferation in lung cancer cells representing different histological subtypes, suggesting a general oncogenic function of LMO1 in lung cancer. In investigating the clinical relevance of LMO1 as an oncogene, we found that a high tumor level of the LMO1 mRNA was an independent predictor of poor patient survival. These results suggest that LMO1 acts as an oncogene, with expression correlated with neuroendocrine differentiation of lung cancer, and that it is a determinant of lung cancer aggressiveness and prognosis. By combining gene expression correlations with patient survival and functional *in vitro* investigations, we further identified TTK as mediating the oncogenic function of LMO1 in lung cancer cells.

## INTRODUCTION

LMO1 belongs to the family of LIM-only domain genes (LMOs) [1–4]. LMOs encode proteins that share similar LIM domain zinc finger structures mediating protein-protein interactions [5]. The LIM domain binds to a wide variety of protein targets, allowing LIM proteins to function in diverse biological processes ranging from transcriptional regulation of gene expression to interaction with the actin cytoskeleton. Members of the LMO family, including LMO1, LMO2, LMO3 and LMO4, have been indicated to play important roles in the tumorigenesis of several types of cancer, including leukemia, breast cancer, and neuroblastoma [6–10]. LMO1, the first member of the family to be discovered, was identified as activated by chromosomal translocation t(11;14)(p13;q11) in a case of T-lineage acute lymphoblastic leukemia (T-ALL) [4]. Since then, the role of LMO1 in the development of leukemias and lymphomas has been characterized *in vivo* in mouse models [2, 11, 12]. More recently, LMO1 has been reported to have an oncogenic role in other types of cancer [13, 14]. In a study of the function of LMO1 in non-small cell lung cancer (NSCLC), Zhang *et al* found that LMO1 was significantly over-expressed in NSCLC specimens relative to normal adjacent tissue, and that over-expression of LMO1 in NSCLC cells promoted cell proliferation, supporting an oncogenic function in NSCLC [15].

Unlike other LMO members, such as LMO2, which is relatively ubiquitous in tissues, LMO1 has been shown to be limited in expression to specific areas of the central nervous system during development [16]. This suggests that dysregulation of LMO1 may be important to the development of cancers of neural origin. In fact, LMO1 was recently identified through a genome-wide association study as an oncogene associated with neuroblastoma [7], a neuroendocrine tumor that occurs in childhood. The association of LMO1 with neuroblastoma suggests the possible involvement of LMO1 in other types of neuroendocrine cancers, such as neuroendocrine lung cancer. Although Zhang, *et al.* investigated the function of LMO1 in NSCLC [15], no study has specifically investigated the role of LMO1 in neuroendocrine lung cancer.

Neuroendocrine lung cancer is traditionally classified as a distinct subset of aggressive lung cancers that share common morphological and histological characteristics. 95% of all neuroendocrine lung cancers are either small cell lung carcinoma (SCLC) or large cell neuroendocrine carcinoma (LCNEC), the most aggressive and lethal subtypes of all lung cancer, with a median survival of only 7-23 months following treatment [17]. Interestingly, recent studies have shown that 10-30% of NSCLC tumors contain neuroendocrine-differentiated cancer cells [18, 19]. Since the majority of neuroendocrine lung cancers are clinically very aggressive, it is speculated that neuroendocrine differentiation of NSCLC may be a hallmark of NSCLC progression towards a more malignant phenotype with poor prognosis [19]. However, the mechanisms of neuroendocrine differentiation of NSCLC remain largely unknown, hindering development of specific and effective treatments.

In this study, we aimed to determine the relationship between LMO1 expression and neuroendocrine differentiation of lung cancer, to further define the oncogenic function of LMO1 in different histological subtypes of lung cancer cells, and to evaluate the clinical relevance of high LMO1 expression in lung cancer patients. We also explored the mechanisms of LMO1 action in lung cancer cells by combining clinical data analysis and *in vitro* functional investigation.

## RESULTS

### LMO1 mRNA level is a marker of neuroendocrine differentiation of lung cancer cells

**Figure 1:**
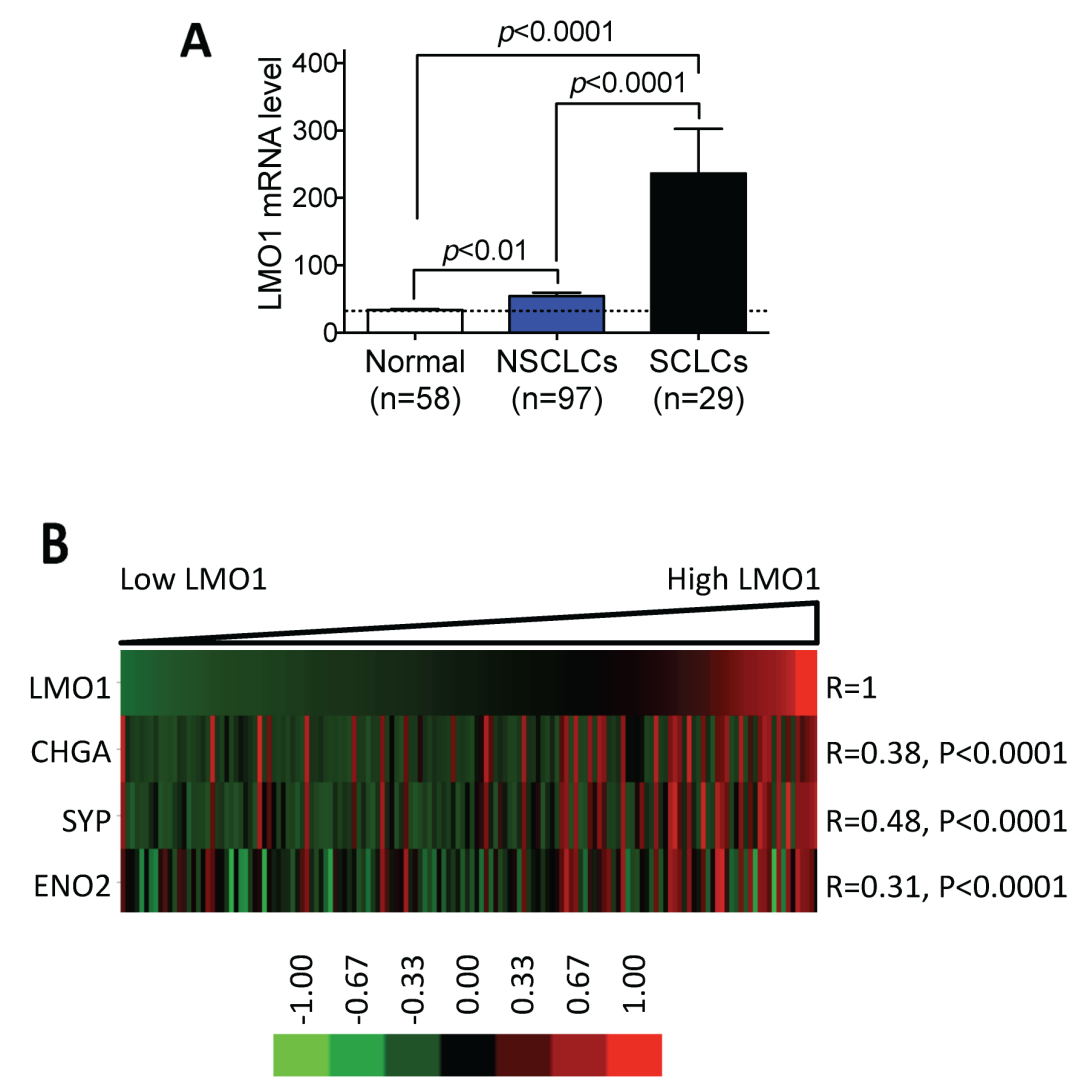
LMO1 mRNA levels are significantly correlated with expression of neuroendocrine markers in lung cancer cell lines **(A)** Comparison of LMO1 mRNA levels in different groups of lung cell lines. LMO1 mRNA levels were measured using Illumina microarrays. Differences in average LMO1 mRNA levels between two groups were assessed using Tukey’s multiple comparison test. **(B)** Correlations between LMO1 mRNA levels and neuroendocrine markers CHGA, SYP, and ENO2. mRNA levels of the indicated genes were measured using Illumina microarrays. Statistical significance of the correlations was assessed by Pearson product-moment correlation coefficient. The heatmap was generated using GenePattern (http://genepattern.broadinstitute.org/gp) and TreeView 3.0. CHGA, chromogranin A; SYP, synaptophysin; ENO2, neuron-specific enolase-2.

To determine the relationship between LMO1 expression and neuroendocrine lung cancer, we analyzed the expression of LMO1 mRNA in a large panel of lung cell lines. The panel of cell lines was classified into three histological groups. As shown in Table 1, the average LMO1 mRNA levels in the three groups were significantly different (*F*=21.13, *p*<0.0001), as assessed by one-way ANOVA. We then used Tukey’s multiple comparison test to examine the differences between the groups. As shown in Table 2 and Figure 1A, LMO1 was expressed at significantly higher levels in SCLC cells relative to both NSCLC cells and immortalized normal lung cells. Although the mean LMO1 mRNA level in NSCLC cells was higher than that in normal cells, the difference between these two groups did not reach statistical significance. These results suggest that elevated LMO1 expression is more closely associated with neuroendocrine lung cancer. To further evaluate the association of LMO1 expression with neuroendocrine differentiation in lung cancer cells, we examined the correlation between LMO1 mRNA levels and mRNA levels of neuroendocrine markers, including chromogranin A (CHGA), synaptophysin (SYP) and neuron-specific enolase-2 (ENO2). These three transcripts have been demonstrated to be markers for neuroendocrine differentiation in lung cancer cells [20–22]. As shown in Figure 1B, LMO1 mRNA levels were significantly correlated with expression of all three neuroendocrine markers. These results suggest that increased LMO1 expression is another potential biomarker for the neuroendocrine differentiation of lung cancer.

**Table 1:** One-way ANOVA analysis of the mean LMO1 mRNA levels in three histological groups of lung cell lines.

Groups	N	Mean	SD	SE	95% CI	Min	Max	*F*	*p* value
Normal	58	34.1	9.8	1.3	31.5-36.6	20.6	62.38	21.13	<0.0001
NSCLC	97	54.6	50.8	5.2	44.3-64.8	20.4	327.4		
SCLC	29	236.3	356.8	66.2	110.6-372.0	20.4	1396.0		

Normal, immortalized Human Bronchial Epithelial Cells (HBECs) and immortalized Human Small Airway Epithelial Cells (HSAECs); NSCLC, non-small cell lung cancer cell lines; SCLC, small cell lung cancer cell lines; SD, standard deviation; SE, standard error; CI, confidence interval; Min, minimum value; Max, maximum value.

**Table 2:** Tukey’s multiple comparison test of mean LMO1 mRNA levels between paired histological groups of lung cell lines.

Groups	Mean diff	95% CI of diff	Adjusted *p* value	Significant?
Normal vs. NSCLC	-20.52	-77.49 to 36.44	0.6715	No
Normal vs. SCLC	-202.2	-280.3 to -124.2	<0.0001	Yes
NSCLCs vs. SCLC	-181.7	-254.3 to -109.1	<0.0001	Yes

LMO1 mRNA levels were measured as above. Diff, difference; CI, confidence interval.

### LMO1 promotes survival and proliferation of lung cancer cells of different histological subtypes and genetic backgrounds

**Figure 2:**
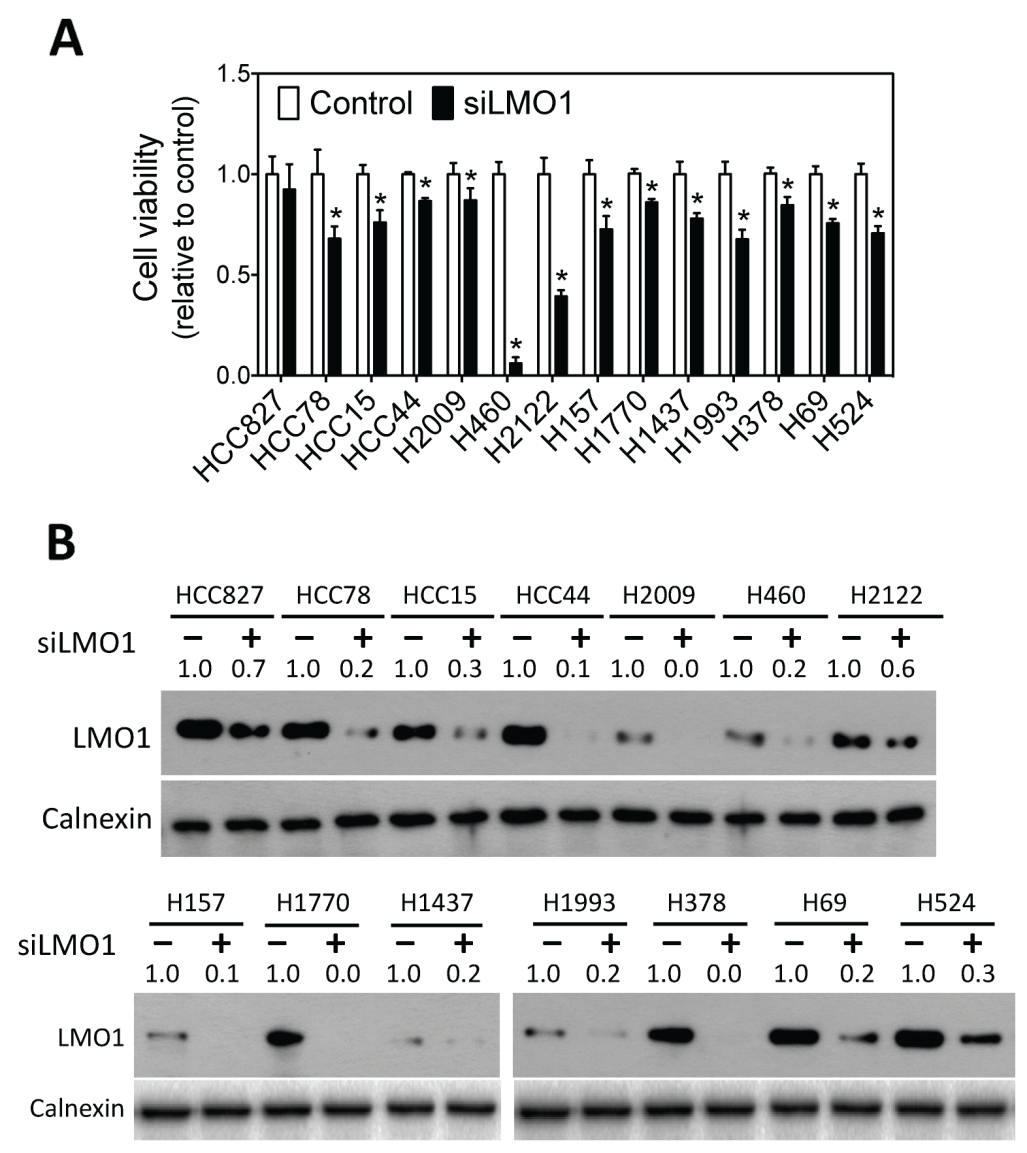
Knock-down of LMO1 expression decreases survival of lung cancer cells **(A)** Effect of LMO1 knock-down on lung cancer cell viability. The indicated lung cancer cell lines were transfected with either 25 nM of siRNA oligos against LMO1 (siLMO1) or negative control oligo (Control) (Dharmacon). Cell viability was measured after 96 h. ^*^, *p*<0.05, relative to control oligo. **(B)** Depletion of LMO1 protein levels by siLMO1. Cells were transfected as above. After 72 h, cell lysates were collected, and LMO1 levels were measured by western blot using mouse anti-LMO1 and rabbit anti-calnexin. Band intensities were quantified using ImageJ.

To examine whether LMO1 has an effect on lung cancer cell survival, we knocked down LMO1 expression in a panel of lung cancer cell lines of different histological subtypes with different genetic backgrounds and various LMO1 mRNA expression levels (Supplementary Table 1). As shown in Figure 2A, knock-down of LMO1 levels by siRNA significantly decreased the survival of 13 of the 14 cell lines tested. The extent of LMO1 protein down-regulation by siLMO1 was confirmed by western blot (Figure 2B). Although three of the cell lines − HCC827, HCC78 and HCC44 − have very low LMO1 mRNA levels (Supplementary Table 1), they expressed Lmo1 protein at levels comparable to some cell lines with high LMO1 mRNA levels, as indicated by western blot. This explains why knock-down of LMO1 in cell lines HCC78 and HCC44 significantly decreased cell survival. The LMO1 protein level in HCC827 was not dramatically depleted by siLMO1, which may explain why siLMO1 did not significantly decrease survival of these cells. LMO1 mRNA levels were also not effectively decreased by siLMO1 (Supplementary Figure 2A), as measured by qRT-PCR, suggesting a low transfection efficiency of siLMO1 in this particular cell line.

**Figure 3:**
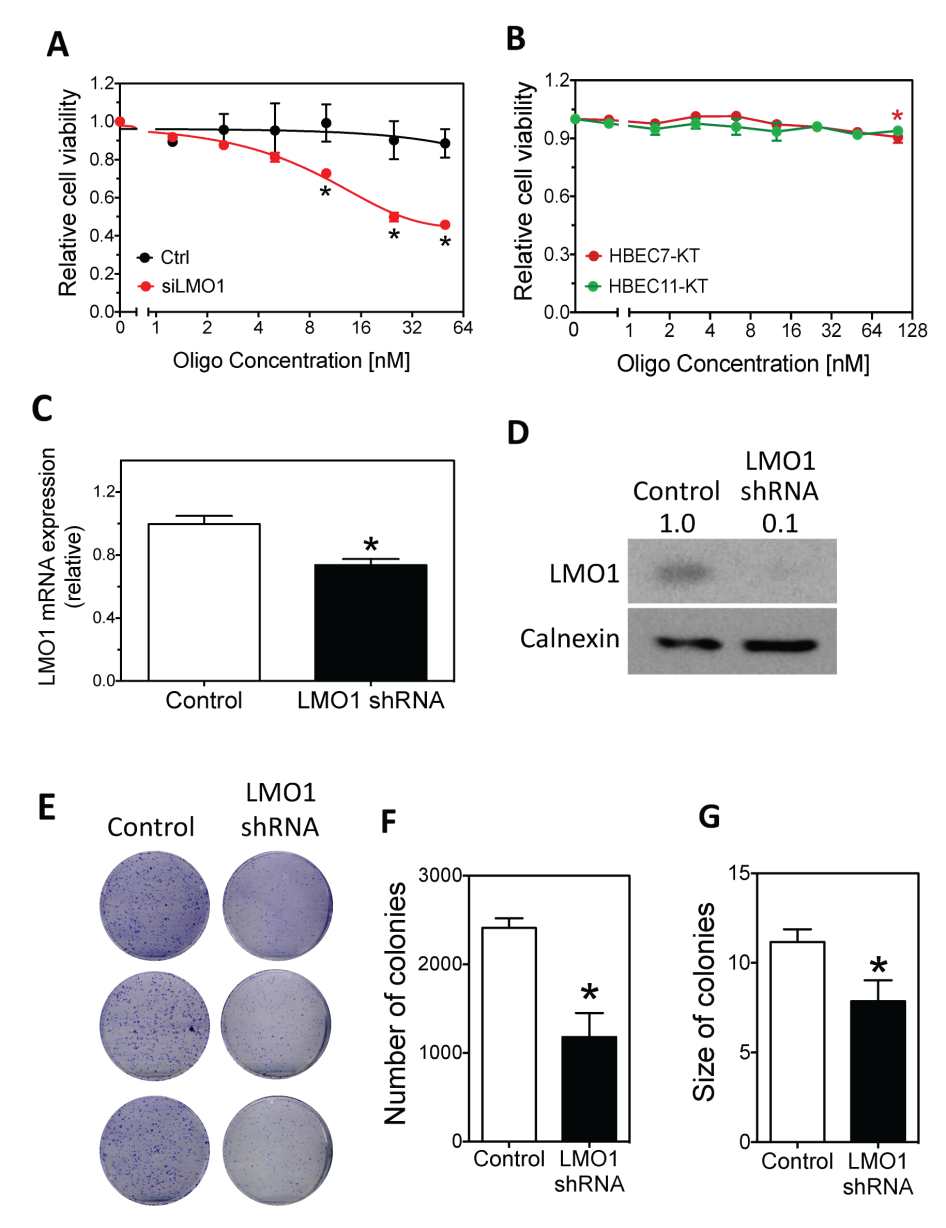
Effect of LMO1 knock-down on cell survival and colony formation in H1993 cells and HBECs **(A)** Dose-dependent effect of LMO1 knock-down by siLMO1 on H1993 cell survival. H1993 cells were transfected with different concentrations of siLMO1 or control oligo. Cell viability was measured after 96 h.^*^, *p*<0.05, relative to control oligo at the same concentration. **(B)** Effect of LMO1 knock-down with siLMO1 on cell viability in HBECs. HBEC-7KT and HBEC-11KT cells were transfected and cell viability was measured as above.^*^, *p*<0.05, relative to mock transfected cells (0 nM). **(C-D)** Depletion of LMO1 mRNA and protein levels using LMO1 shRNA. H1993 cells with stably integrated LMO1 shRNA or control shRNA were established as described in Materials and Methods. RNA was isolated for measuring mRNA levels by qRT-PCR (C) and protein lysates were collected for measuring protein levels by western blot as above (D). ^*^, *p*<0.05, relative to control oligo. **(E-F)** Colony formation and quantification of colonies as function of LMO1 knock-down with LMO1 shRNA. ^*^, *p*<0.05, relative to control.

To compare the effect of LMO1 depletion on survival of lung cancer cells to that of normal lung cells, we transfected increasing doses of siLMO1 and assessed survival of lung cancer line H1993 and immortalized normal HBEC cells. As shown in Figure 3A, siLMO1 decreased H1993 cell viability in a dose-dependent manner. In contrast, as shown in Figure 3B, siLMO1 at concentrations ≤ 50nM did not significantly decrease cell viability in the HBEC cells; it only slightly but significantly decreased the survival of one HBEC (HBEC7-KT) at a high concentration (100 nM). The extent of LMO1 mRNA knock-down by siLMO1 in HBECs was comparable to that in H1993 cells (Supplementary Figure 2B), as measured by qRT-PCR, excluding the possibility that the lack of response of HBECs to siLMO1 treatment was due to low transfection efficiency. Coupled with the much higher expression of LMO1 in H1993 cells than in HBEC7-KT and HBEC11-KT cells (Supplementary Table 1), the differential response to LMO1 knock-down between H1993 and HBECs suggests that LMO1 expression is important for maintaining the survival of lung cancer cells with high LMO1 expression but not for normal HBECs with low LMO1 expression.

**Figure 4:**
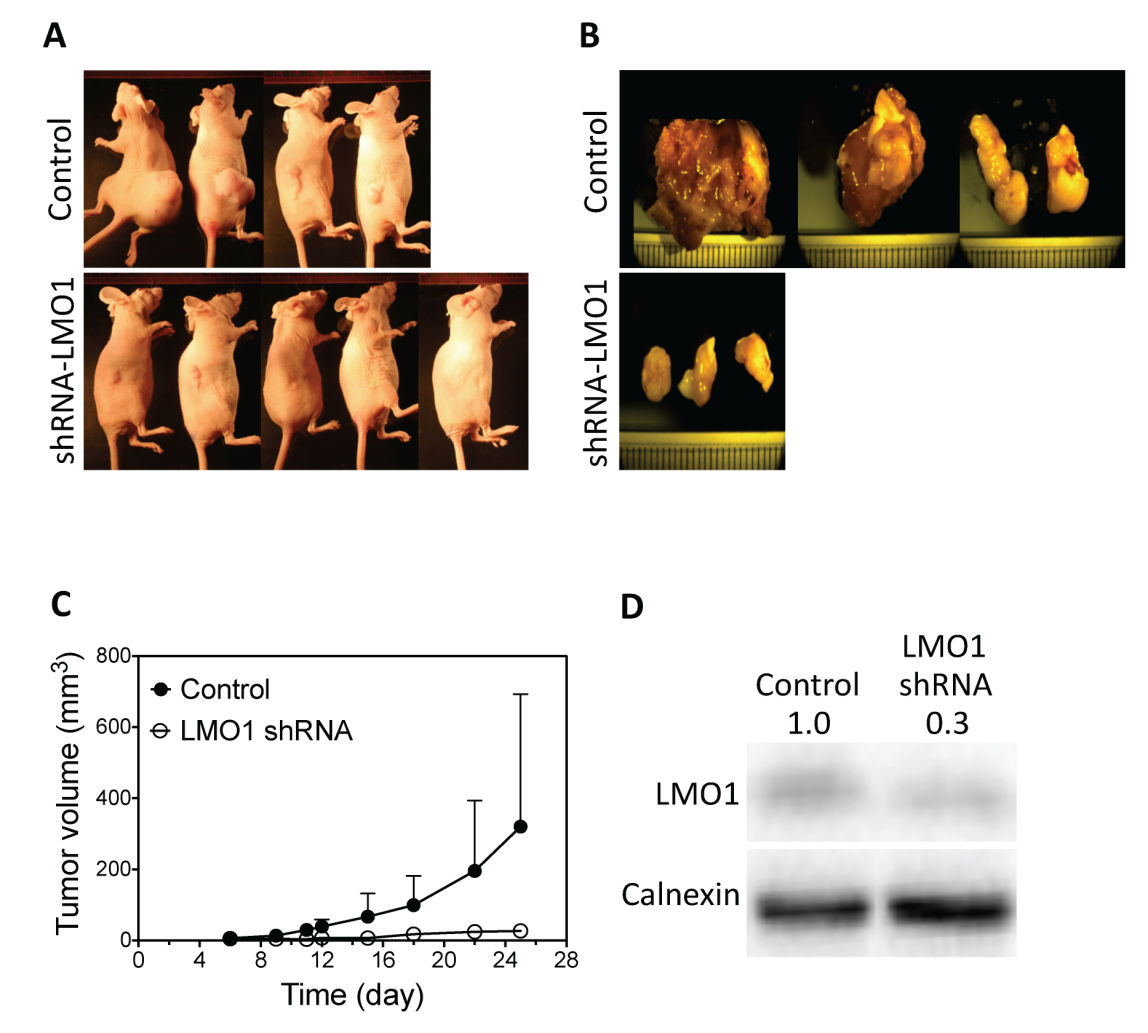
LMO1 knock-down inhibits tumor growth in mouse lung tumor xenografts **(A)** Images of mice injected with LMO1 shRNA H1993 cells or control-shRNA H1993 cells (Control). Images were taken at the end point of the experiment. **(B)** Tumor images from each group. **(C)** Tumor growth curves from each group. **(D)** LMO1 protein levels in tumors as measured by western blot.

We next examined the long-term effect of LMO1 depletion on H1993 cell proliferation through colony formation assays by stably knocking down expression of LMO1 by shRNA. The depletion of LMO1 mRNA and protein were confirmed by qPCR and western blot, respectively (Figure 3C–3D). Figure 3E–3G shows that LMO1 depletion by shRNA significantly reduced the capacity of H1993 cells to form colonies, reducing both colony sizes and numbers. To preliminarily examine the function of LMO1 *in vivo*, we generated a mouse lung cancer xenograft model using the engineered H1993 cell lines described above. As shown in Figure 4A–4B, only three of the five mice in the shRNA-LMO1 group grew tumors, whereas all four mice in the control group grew tumors. In addition, the average tumor growth rate in the shRNA-LMO1 group was much slower than in the control group, although the difference in tumor size at each time point did not reach statistical significance, likely attributable to the small sample size (Figure 4C). LMO1 protein levels in the tumor tissues were consistent with those observed in the engineered H1993 cell lines as assessed by western blot, shown in Figure 4D.

**Figure 5:**
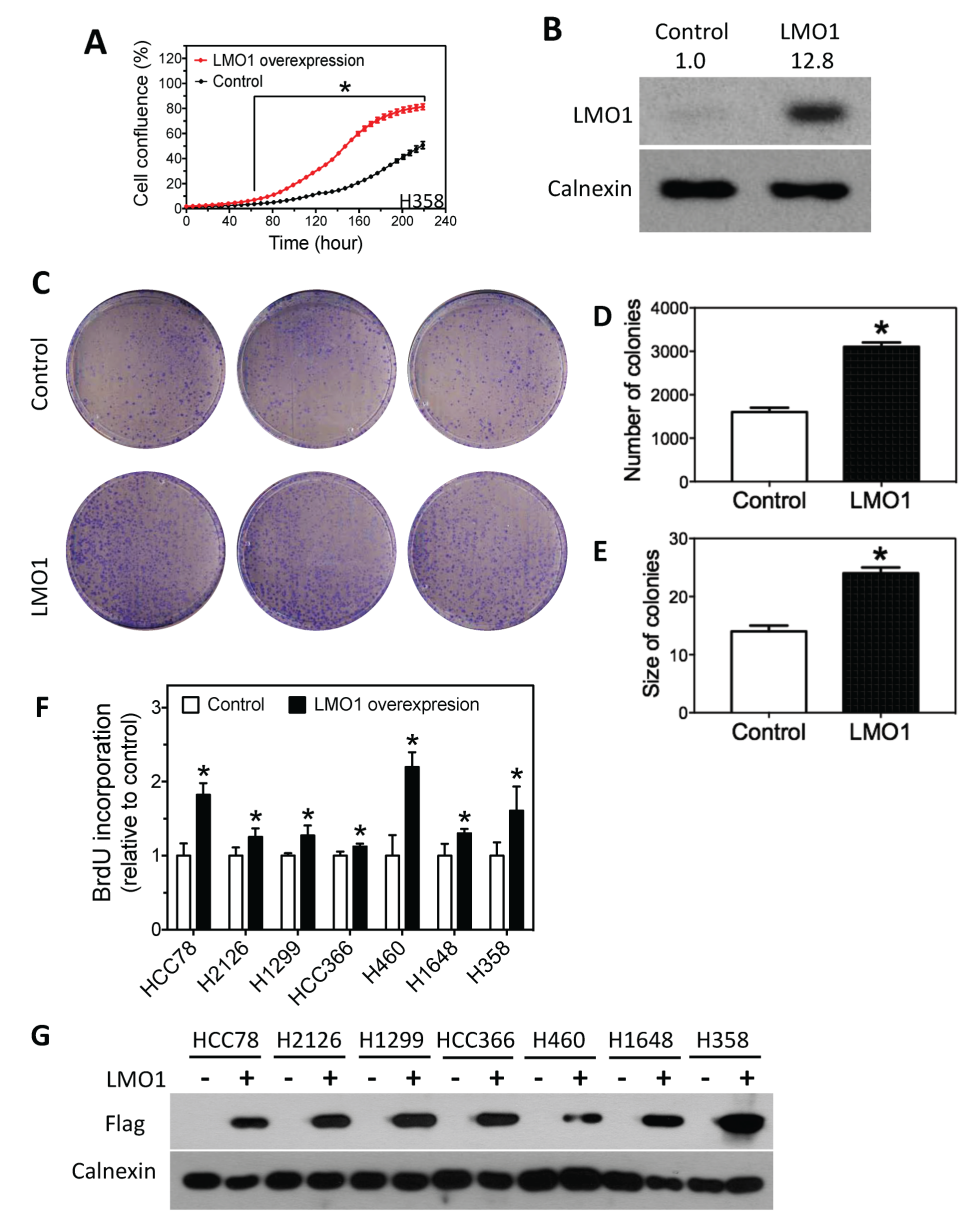
Over-expression of LMO1 promotes lung cancer cell proliferation **(A)** Cell confluence as a function of time. H358 cells were transfected with either pcDNA3.1-Flag-LMO1 or empty pcDNA3.1 vector (control), and cell confluence was measured over 9 days. The statistical significance of difference in confluence between LMO1 over-expression and control at each time point was determined by student’s *t*-test. ^*^, *p*<0.05, relative to control. **(B)** Over-expression of LMO1 protein levels by pcDNA3.1-Flag-LMO1 in H358 cells. Cells were transfected with either pcDNA3.1-Flag-LMO1 (LMO1) or pcDNA3.1 (Control). After 72 h, protein lysates were collected for measuring protein levels by western blot. **(C-E)** Colony formation and quantification of colonies as a function of LMO1 over-expression. **(F)** BrdU incorporation as a function of LMO1 over-expression in a panel of lung cancer cell lines. **(G)** Over-expression of LMO1 protein was detected by anti-Flag antibody. ^*^, *p*<0.05, relative to control.

To further characterize the growth-promoting function of LMO1 in lung cancer cells, we examined the effect of LMO1 over-expression in H358 cells, a lung cancer cell line with low endogenous levels of LMO1 mRNA (Supplementary Table 1). As shown in Figure 5A, LMO1 over-expression via expression vector pcDNA3.1-Flag-LMO1 dramatically promoted cell proliferation, as measured by the change in cell confluence over time, compared to controls: confluence of the LMO1-overexpressing cells was significantly higher than that of control cells 60 h after transfection with pcDNA3.1-Flag-LMO1. The extent of LMO1 protein over-expression by pcDNA3.1-Flag-LMO1 was confirmed by western blot (Figure 5B). As expected, LMO1 over-expression significantly enhanced the capacity of H358 cells to form colonies, resulting in both increased colony sizes and numbers (Figure 5C–5E). Furthermore, LMO1 over-expression increased cell proliferation in 7 lung cancer cell lines with low or medium LMO1 mRNA levels, as measured by BrdU incorporation (Figure 5F). As above, the extent of LMO1 protein over-expression by pcDNA3.1-Flag-LMO1 was confirmed by western blot (Figure 5G). Together, these results indicate that LMO1 functions to promote survival and proliferation of lung cancer cells.

### Lung tumor level of LMO1 mRNA is an independent predictor of patient survival

**Figure 6:**
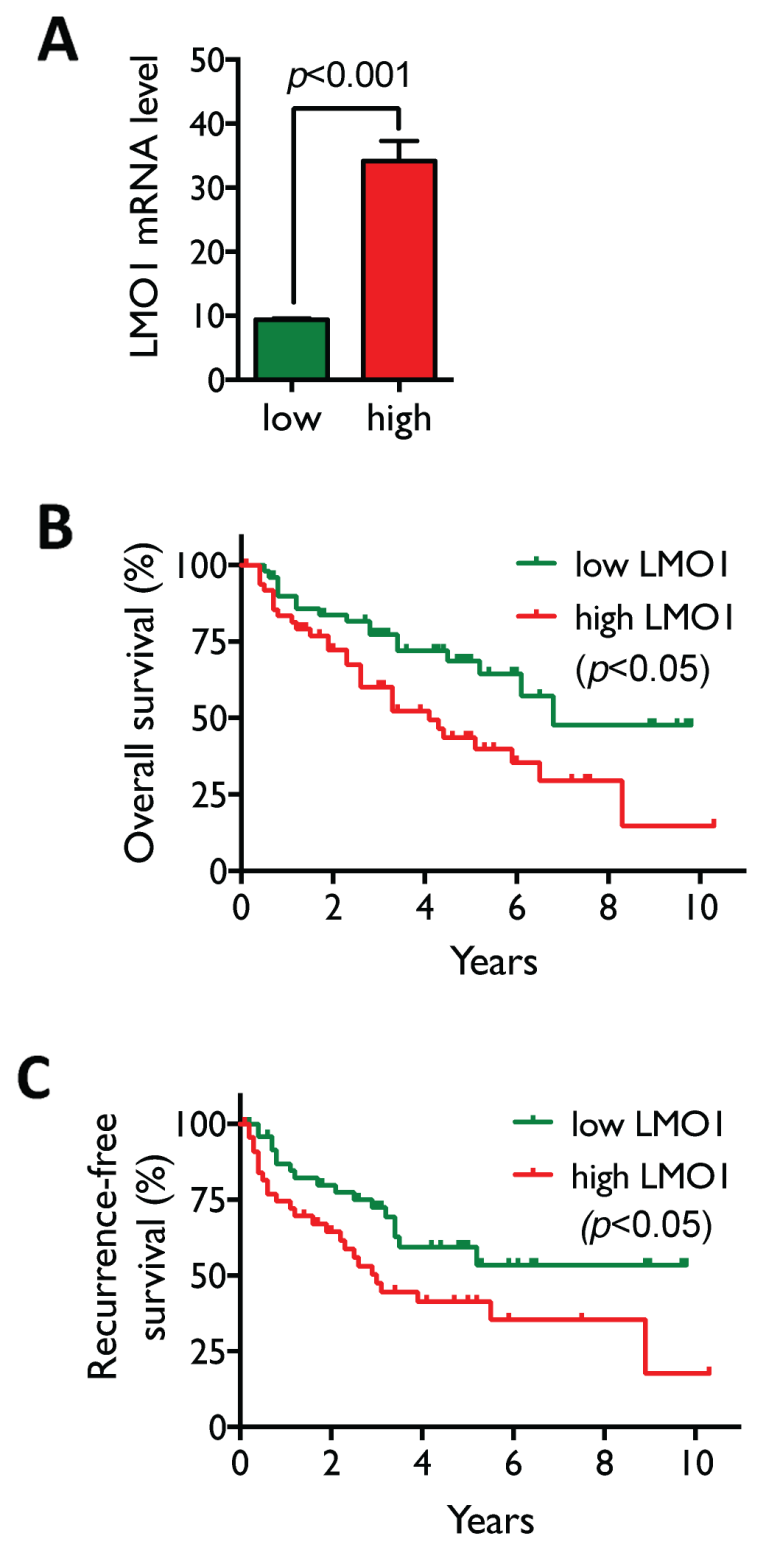
High LMO1 levels correlate with poor survival of lung cancer patients **(A)** Patients with LMO1 mRNA levels in the highest and lowest quintiles were selected from the 245 NSCLC patients in the MDACC dataset to define the high and low LMO1 expression groups (n=50/group). Statistical significance of the difference in mRNA levels between the two groups was assessed by Student’s *t*-test. **(B-C)** Statistical significance of differences in overall (B) and recurrence-free survival (C) were assessed by Mantel-Cox log-rank test.

To examine the clinical relevance of LMO1 expression in lung cancers, we first assessed the correlation between LMO1 mRNA levels and patient survival by Kaplan-Meier analysis in a dataset of 245 NSCLC lung cancer patients collected at MD Anderson Cancer Center (MDACC). The high and low expression groups were defined as described in the Figure 6 legend. As shown in Figure 6A, LMO1 mRNA levels in the high LMO1 patient group were significantly higher than those in the low LMO1 group. Median overall survival times in the high and low LMO1 groups were 4.1 y and 6.8 y, respectively, with a hazard ratio of 2.11 (95% CI 1.17-3.84), and *p*<0.05 (Figure 6B). Median recurrence-free survival times in the high and low LMO1 groups were 3.0 years and >10 y, respectively, with a hazard ratio of 1.99 (95% CI 1.07-3.68), and *p*<0.05 (Figure 6C).

To further examine whether LMO1 is an independent predictor of patient survival, we investigated the relationship between LMO1 mRNA expression and other clinicopathological features of the disease by multivariate analysis in two datasets, the above-mentioned MDACC dataset and the Director’s Challenge Adenocarcinoma Lung Study dataset [23, 24]. The characteristics of the patients are shown in Supplementary Table 2. The stratification of the six variables used in the multivariate analysis is shown in Supplementary Table 3. As shown in Table 3A, when analyzing the whole patient population in each of the two dataset, LMO1 mRNA level was not identified as an independent predictor of lung cancer patient survival. We conducted further analyses after stratifying both datasets. Interestingly, as shown in Table 3B, LMO1 was identified as an independent predictor in both datasets in a specific patient subgroup, the stage I-II Caucasian adenocarcinoma patients without adjuvant or neoadjuvant chemotherapy. Altogether, our results suggest that LMO1 expression is an independent determinant of lung cancer progression and prognosis at least in specific subgroups of patients. As expected, patient age and disease stages, parameters well recognized as predictors of patient survival in many types of cancers, were identified as independent predictors of lung cancer patient survival in at least one of the two datasets (Table 3A-3B). Tobacco usage was not identified as an independent predictor in our analyses, mostly because of the small variations in tobacco usage in the datasets that we analyzed.

**Table 3:** Multivariate analysis of clinicopathological parameters.

(A) Analysis of unfiltered datasets.				
	MDACC Dataset (n=245)		Director’s Challenge Dataset (n=440)	
Parameter	HR (95% CI)	*p*	HR (95% CI)	*p*
LMO1	1.01 (0.99-1.02)	0.300	1.01 (1.00-1.02)	0.094
Age	1.02 (1.00-1.04)	0.056^*^	1.03 (1.02-1.04)	2.56E-05^***^
Gender	1.44 (0.94-2.21)	0.094	1.24 (0.94-1.62)	0.126
Tobacco	0.78 (0.55-1.10)	0.154	0.96 (0.80-1.15)	0.680
T stage	1.12 (0.90-1.40)	0.317	1.53 (1.27-1.85)	7.00E-06^***^
N stage	1.67 (1.31-2.13)	2.99E-05^***^	1.86 (1.59-2.17)	5.00E-15^***^

(B) Analysis of filtered datasets.				
	MDACC Dataset (n=67)		Director’s Challenge Dataset (n=138)	
Parameter	HR (95% CI)	*p*	HR (95% CI)	*p*
LMO1	1.11 (1.03-1.19)	0.007^**^	1.03 (1.00-1.05)	0.027^*^
Age	1.08 (1.03-1.14)	0.003^**^	1.06 (1.02-1.09)	0.001^***^
Gender	1.08 (0.45-2.59)	0.865	0.93 (0.51-1.69)	0.803
Tobacco	0.90 (0.43-1.91)	0.788	1.44 (0.82-2.50)	0.201
T stage	1.26 (0.45-3.48)	0.659	1.54 (0.84-2.84)	0.164
N stage	0.80 (0.30-2.12)	0.651	2.06 (0.96-4.43)	0.063

Correlations of multiple clinicopathological parameters with lung cancer patient survival were assessed using multivariate Cox proportional-hazards regression. Shown are analyses of **(A)** unfiltered patient datasets and **(B)** datasets filtered for stage I-II Caucasian adenocarcinoma patients without adjuvant or neoadjuvant chemotherapy. HR, Hazard Ratio; T stage, disease stage based on tumor size; N stage, disease stage based on the degree of spread to lymph nodes. ^*^, *p*<0.05; ^**^, *p*<0.01; ^***^, *p*<0.001.

### Candidate genes mediating the function of LMO1 are identified by combined analyses of gene expression correlations and lung cancer patient survival

We next sought to determine the gene(s) that mediate the function of LMO1 in lung cancer. In order to directly identify candidate mediators that are relevant to both lung cancer tumorigenesis and patient survival, we began with genes displaying significant correlation with mRNA levels of LMO1 and neuroendocrine markers CHGA, SYP and ENO2 levels in the MDACC dataset. From these genes, we narrowed the set of candidates to those with mRNA expression significantly higher in tumor tissue than in normal adjacent tissue (NAT), and with expression levels significantly correlated with patient survival. We identified six genes (Table 4 and Supplementary Table 4), including GNG4 (encodes G protein subunit gamma 4, NCAPG (encodes non-SMC condensin I complex subunit G protein), SPC25 (encodes kinetochore protein SPC25), TTK (encodes protein kinase TTK), ASPM (encodes abnormal spindle-like microcephaly-associated protein), KIF2C (encodes kinesin family member 2C protein), with mRNA expression levels significantly correlated with mRNA levels of LMO1 and two or more neuroendocrine markers, with high expression correlated with poor patient survival (Supplementary Figure 1), and with expression in tumor tissue significantly higher than in NAT.

**Table 4:** Candidate genes identified as potentially mediating the function of LMO1 in lung cancer.

(a) Symbol	(b) Correlation (R)				(c) Rstat	(d) Ostat	(e) Tumor	(f) Normal	(g) Ratio
	LMO1	CHGA	SYP	ENO2					
GNG4	0.24^***^	0.39^***^	0.33^***^	0.20^***^	1.69	11.81^*^	234	34	6.9
NCAPG	0.27^***^	0.19^***^	0.00	0.24^***^	4.54^*^	7.09^*^	838	152	5.5
SPC25	0.26^***^	0.18^***^	0.01	0.15^***^	6.80^*^	12.27^*^	385	65	5.9
TTK	0.24^***^	0.16^***^	-0.06	0.25^***^	6.59^*^	11.08^*^	685	129	5.3
ASPM	0.24^***^	0.15^***^	0.00	0.14^***^	4.14^*^	6.65^*^	838	143	5.9
KIF2C	0.25^***^	0.15^***^	0.01	0.26^***^	3.10	7.10^*^	917	160	5.7

Shown are **(a)** Gene symbol, **(b)** the correlation R values of LMO1, CHGA, SYP and ENO2 mRNA levels with the mRNA levels of the indicated genes, **(c-d)** log-rank chi-square values for comparing recurrence-free survival (Rstat) and overall survival (Ostat) between the high and low LMO1 groups, **(e-f)** average mRNA expression levels of the indicated genes in tumors **(e)** and normal adjacent tissues **(f)**, and **(g)** the tumor *vs.* normal ratio. Results were based on the MDACC dataset. Rstat > 3.84 and Ostat > 3.84 indicate that high LMO1 mRNA levels are significantly correlated with poor recurrence-free and overall survival, respectively. ^*^, *p*<0.05; ^***^, *p*<0.001.

### TTK plays a role in mediating the function of LMO1 in lung cancer cells

**Figure 7:**
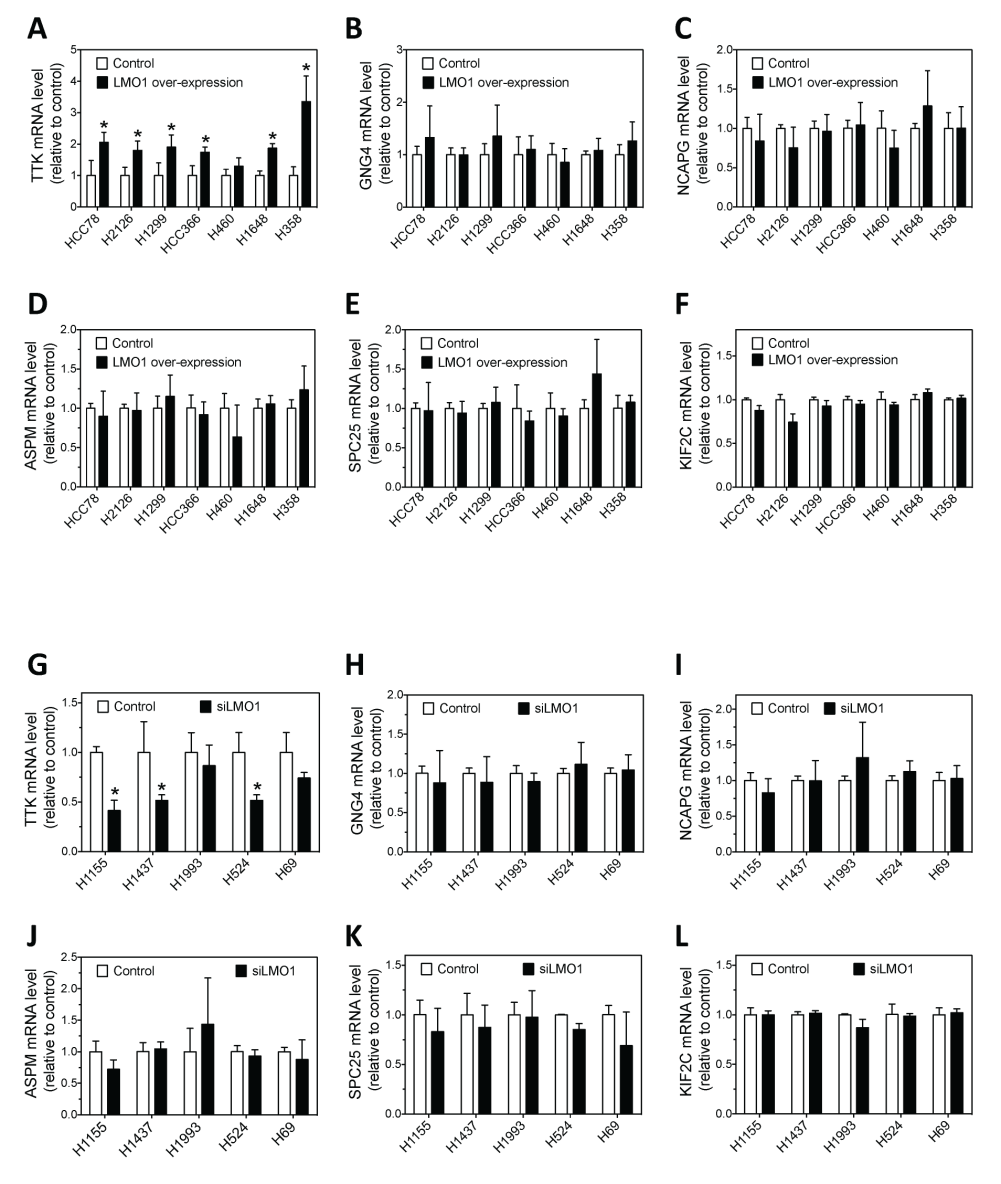
Effect of LMO1 over-expression and knock-down on mRNA expression levels of the six candidate genes in lung cancer cells **(A-F)** Effect of LMO1 over-expression on mRNA expression of TTK, GNG4, NCAPG, ASPM, SPC25 and KIF2C in a panel of cell lines. Each cell line was transfected with either pcDNA3.1-Flag-LMO1 or control vector. After 72 h, RNA was isolated from cells and mRNA levels were measured as above. **(G-L)** Effect of LMO1 knock-down on mRNA expression of TTK, GNG4, NCAPG, ASPM, SPC25 and KIF2C in a panel of cell lines. Each cell line was transfected with either siLMO1 or control oligo (control). After 72 h, mRNA levels were measured as above. ^*^, *p*<0.05, relative to control.

We investigated whether LMO1 regulates the expression of the above six candidate genes in multiple lung cancer cell lines. As shown in Figure 7A, over-expression of LMO1 with pcDNA3.1-Flag-LMO1 expression vector significantly up-regulated expression of TTK mRNA as compared with cells transfected with empty vector pcDNA3.1 in six of the seven lung cancer cell lines tested. Only in H460 cells did the up-regulation not reach statistical significance. Figure 7G further shows that knock-down of LMO1 expression by siLMO1 decreased expression of TTK mRNA levels in all five of the cell lines tested, although the differences in two cell lines, H1993 and H69 did not reach statistical significance. These results indicate LMO1 acts to up-regulate expression of TTK in lung cancer cells. Investigation of the remaining five candidate genes (GNG4, NCAPG, ASPM, SPC25 and KIF2C) indicated that LMO1 does not play a significant role in regulating their expression in lung cancer cells, as shown in Figure 7B–7F and 7H–7L. Overall, the above results indicates that, among the six candidate mediators of LMO1 function that we identified, LMO1 only regulates TTK expression in lung cancer cells.

**Figure 8:**
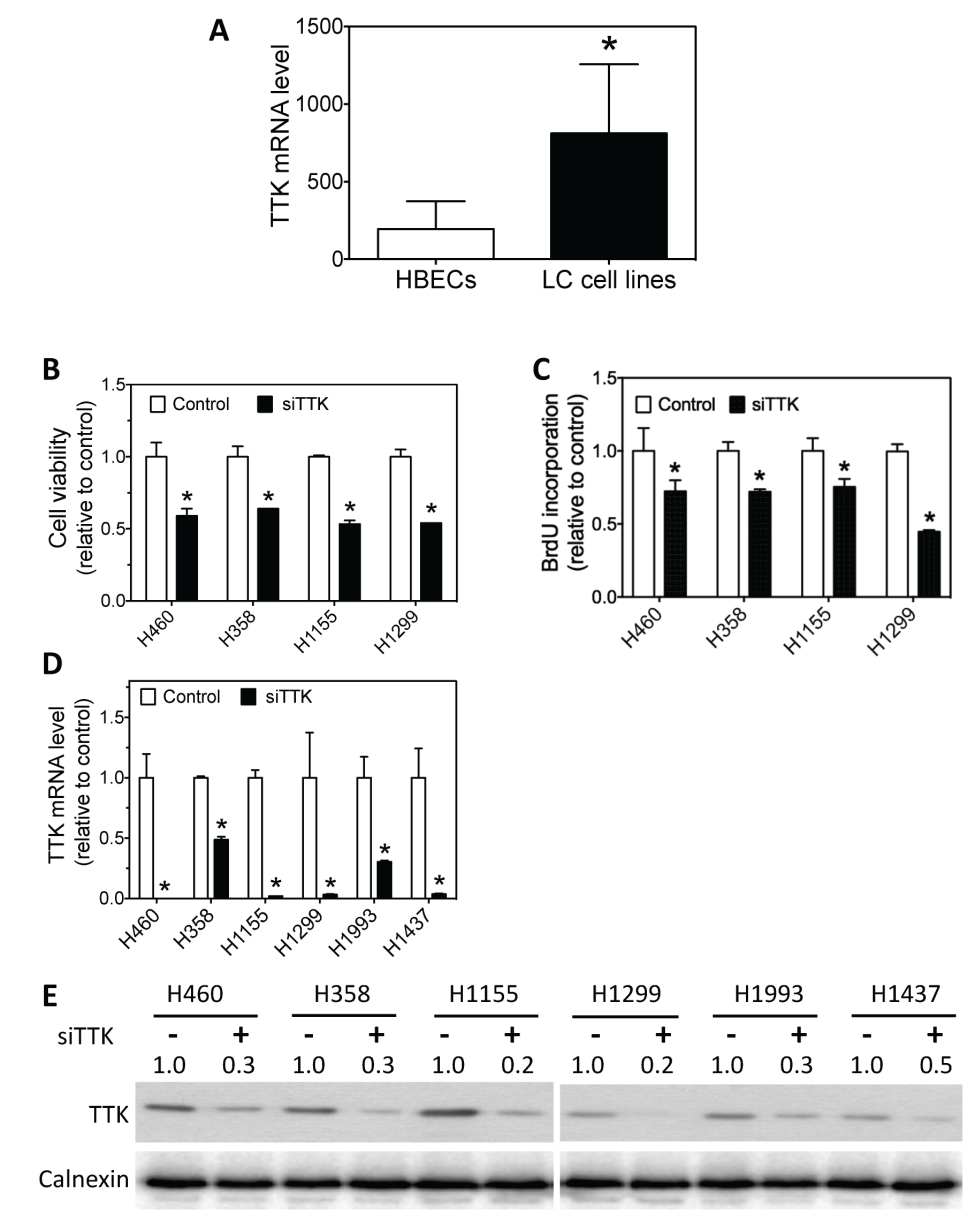
Lung cancer cells have higher TTK mRNA levels than HBECs, and knock-down of TTK expression reduces lung cancer cell survival and proliferation **(A)** Average mRNA expression levels of TTK in a panel of lung cancer (LC) cell lines and a panel of HBECs. Differences in average TTK mRNA levels between the two groups were analyzed using two-tailed *t*-test. ^*^, *p*<0.05, relative to HBECs. **(B-C)** Effect of TTK knock-down on lung cancer cell viability and proliferation. The indicated lung cancer cell lines were transfected with either 25 nM TTK siRNA oligos (siTTK) or negative control oligo (control). After 96 h, cell viability (B) and BrdU incorporation (C) were measured as above. ^*^, *p*<0.05, relative to control oligo. **(D-E)** Depletion of TTK mRNA and protein levels using siTTK. Cells were transfected as above. After 72 h, TTK mRNA (D) and protein levels (E) were measured as above. ^*^, *p*<0.05, relative to control oligo.

Consistent with the observed over-expression in lung tumor specimens relative to NATs (Table 4), our investigation in a panel of cell lines showed that TTK mRNA was over-expressed in lung cancer cell lines relative to immortalized normal HBEC cells (Figure 8A). We then examined whether TTK knock-down affects lung cancer cell survival and proliferation. As shown in Figure 8B, knock-down of TTK expression by siTTK significantly decreases lung cancer cell survival. Figure 8C further shows that siTTK also significantly decreases cell proliferation as measured by BrdU incorporation. The depletion of TTK mRNA and protein levels by siTTK were confirmed by qRT-PCR and western blot, as shown in Figure 8D–8E.

Taken together, these results suggest that TTK plays a key role in mediating the oncogenic function of LMO1 in lung cancer cells.

## DISCUSSION

In this study, we identified LMO1 as a potential biomarker of neuroendocrine differentiation of lung cancer. We found that LMO1 mRNA levels are significantly correlated with mRNA levels of neuroendocrine markers, CHGA, SYP and ENO2. These findings may be especially important for NSCLC with the propensity for neuroendocrine differentiation. Studies have shown that around 10-30% of NSCLCs contain neuroendocrine-differentiated cancer cells [18, 19]. Since the majority of neuroendocrine lung cancers are clinically very aggressive, it has been speculated that neuroendocrine differentiation of NSCLC may be a hallmark of NSCLC progression towards a more malignant phenotype with poor prognosis [19]. Indeed, several studies have shown that neuroendocrine differentiation of NSCLC is associated with poor patient survival [25–27]. However, contradictory results were observed in other studies [26, 28]. The inconsistency between these observations is believed to be attributable to differences in the diagnostic markers used to define neuroendocrine differentiation [18, 19]. Several protein markers, including CHGA, SYP and ENO2, associated with neuroendocrine lung cancer have been investigated as diagnostic markers for NSCLC neuroendocrine differentiation [29, 30]. However, the known neuroendocrine markers seem to be merely non-oncogenic correlates of neuroendocrine differentiation, and have not been found to function as regulators of lung cancer cell growth. This may explain why these markers are not reliable predictors of cancer aggressiveness and patient prognosis. Identifying the neuroendocrine-specific oncogenic pathways that promote the aggressive growth of neuroendocrine-differentiated lung cancer cells can help to develop more reliable diagnostic and prognostic markers for neuroendocrine differentiation in NSCLC. Here, we report that LMO1 expression is not only significantly correlated with expression of all three neuroendocrine markers in NSCLC but also an independent predictor of patient survival. Coupled with our *in vitro* findings that LMO1 functions to promote growth of lung cancer cells, our results support LMO1 expression as a functional oncogenic and prognostic biomarker for neuroendocrine differentiation of NSCLC.

In this study, our multiple-sample statistical analysis of the LMO1 mRNA levels between the three histological cell line groups showed that the difference of LMO1 mRNA levels between NSCLC and normal cells did not reach statistical significance, which is inconsistent with the report by Zhang et al. [15]. This apparent inconsistency can be fully explained by the different statistical approaches that we exploited. Zhang et al. used two-sample comparisons to assess the difference between NSCLC and normal adjacent tissues [15], which is valid for a study that only includes two groups. Indeed, our use of a *t*-test to compare the NSCLC and normal cell groups also shows that the LMO1 levels in NSCLCs are significantly higher than in normal cells (Supplementary Table 5), consistent with the findings by Zhang et al. Our study, however, is meant to determine whether the SCLC group stands out as a statistically distinct group among the three groups in terms of LMO1 expression, a question properly addressed by multiple-sample one-way ANOVA and Tukey’s multiple comparison tests.

To define the oncogenic function of LMO1 in lung cancers, we investigated the regulation of cell survival and proliferation by LMO1 in a panel of lung cancer cell lines that were derived from different histological subtypes of lung cancer, including adenocarcinoma, squamous cell carcinoma, small cell lung carcinoma, and large cell neuroendocrine lung carcinoma. We demonstrated that LMO1 has a general cell growth-promoting function independent of the histological subtypes and genetic backgrounds of the lung cancer cells. We also investigated the tumorigenic function of LMO1 *in vivo* in a mouse lung cancer xenograft model generated using H1993 cells. Although the sample size is small, our results clearly show the capacity of LMO1 to promote tumorigenesis. Further studies in genetic mouse models with larger sample sizes are certainly needed in order to fully understand the role of LMO1 in lung cancer tumorigenesis.

In multivariate analysis of the determinants of patient survival, we identified LMO1 as an independent predictor in Caucasian patients with stage I or stage II adenocarcinoma without adjuvant or neoadjuvant chemotherapy. These results suggest that LMO1 may be a more critical determinant of survival in patients with early stage disease. This is certainly interesting, and needs to be confirmed in additional clinical investigation.

In order to identify the mechanisms underlying the oncogenic function of LMO1 and its direct clinical relevance, we exploited clinical data from lung cancer patients. Our analyses identified six candidate genes that are significantly correlated with LMO1 expression, significantly up-regulated in lung cancer specimens and significantly correlated with poor patient survival. However, our *in vitro* investigations showed that LMO1 controlled only TTK expression, and did not regulate expression of the other five candidates. Further investigation showed that TTK functions to promote cell survival and proliferation. Together, these results support a role for TTK in mediating the function of LMO1 in lung cancer cells. TTK (also known as MPS1), a dual-specificity protein kinase with the ability to phosphorylate tyrosine, serine and threonine residues [31, 32], plays an important role in controlling centrosome duplication and accurate segregation of chromosomes during mitosis [33, 34]. The oncogenic function of TTK has been well demonstrated, and TTK inhibitors have been used to treat cancers [35–37]. We provide the first identification that TTK acts as a downstream mediator of LMO1 function in lung cancer cells. The mechanisms by which LMO1 regulate TTK expression, however, need to be defined in future studies. While LMO1 is a transcription factor, it has no direct DNA-binding activity and it functions as a transcriptional regulator through LIM-domain interaction with other proteins that bind DNA [12, 38, 39]. Several transcription factors, including OLIG2 [39] and SCL [38], have been shown to interact with LMO1. However, given the large spectrum of interactions of other LMO family members such as LMO2 [40, 41], it is plausible to speculate that the “interactome” and “transcriptome” of LMO1 remain largely undiscovered. The question of which binding partners of LMO1 are involved in regulating TTK expression needs to be further investigated.

In addition to our findings on LMO1, we identified five genes (GNG4, NCAPG, SPC25, ASPM and KIF2C) that are expressed at higher levels in lung tumors relative to NATs and are significantly correlated with poor survival of lung cancer patients, suggesting possible oncogenic functions in lung cancer. However, their roles in lung cancer development have not been investigated. Although our results do not support their roles in mediating the function of LMO1, their potential oncogenic functions certainly warrant further pursuit.

Another interesting finding of our study is that LMO1 protein levels were not closely correlated with LMO1 mRNA levels across the panel of cell lines. Three cell lines (HCC827, HCC78 and HCC44) have very low LMO1 mRNA levels. Their LMO1 protein levels, however, are very high and comparable to the three cell lines with high LMO1 mRNA levels (H524, H69 and H378). These results suggest that post-transcriptional and/or post translational mechanisms may play an important role in determining the LMO1 protein expression levels in at least some, if not all, lung cancers. It also implies that LMO1 protein levels might be a more reliable marker for predicting neuroendocrine differentiation and patient survival than mRNA levels of LMO1 and other neuroendocrine markers − this certainly deserves further investigation.

Overall, our work provides new insights into the function of LMO1 in lung cancer. We showed for the first time that LMO1 has the general property of promoting cell proliferation in lung cancer cells representing different histological subtypes. We discovered a correlation between LMO1 expression and both neuroendocrine differentiation and lung cancer patient survival. In addition, we demonstrated for the first time the involvement of TTK in mediating the oncogenic function of LMO1. This evidence points to an important role for LMO1 in the tumorigenesis of lung cancer. Future studies are certainly warranted to further define the molecular pathways by which LMO1 regulates TTK expression, and to further evaluate the clinical significance of LMO1 expression as a prognostic marker of neuroendocrine differentiation of lung cancers and patient survival in prospective studies.

## MATERIALS AND METHODS

### Cell lines

The cell lines used for the Illumina gene expression microarrays, including 97 NSCLC lines, 29 SCLC lines and 58 immortalized normal lines (29 HBECs and 29 HSAECs), and the cell lines used in the *in vitro* and *in vivo* studies, were derived from lung tumor specimens and/or normal lung tissues of lung cancer patients with different genetic backgrounds at the Hamon Center for Therapeutic Oncology Research at UT Southwestern Medical Center at Dallas (UTSW). Cell lines were maintained in RPMI-1640 medium (Life Technologies, Carlsbad, CA) supplemented with 5% fetal bovine serum (Atlanta Biologicals, Lawrenceville, GA). Cell lines were DNA fingerprinted using the GenePrint PowerPlex 1.2 system (Promega, Madison, WI) and confirmed against fingerprint libraries maintained by ATCC and the Minna/Gazdar laboratories at UT Southwestern Medical Center at Dallas.

### Construction of expression vectors

For the LMO1 expression construct, the coding region of the LMO1 mRNA was amplified by PCR from a human cDNA clone (Open Biosystems, Cat. No. EHS1001-98075481). The amplified sequence was inserted into the EcoRI and XhoI restriction sites of the multiple cloning site of expression vector pcDNA 3.1-(n)-Flag (Invitrogen). The coding region of the TTK mRNA was amplified by PCR from a human cDNA clone (Thermo Fisher Scientific, Cat. No. MHS6278-202808350). The amplified sequence was inserted into the BamHI and XhoI sites of the pcDNA3.1+ vector.

### Generation of LMO1-shRNA stably integrated cells

The pGFP-V-RS-LMO1-shRNA (Origene, Cat. No. TG311708) was co-transfected with pAmpho (Clontech) and psPAX2 (Addgene) into 293FT cells (Invitrogen/Life Technologies). Virus-containing media was collected, filtered and added to target cells for infection in the presence of polybrene. Puromycin was then used to select stable LMO1-shRNA integrants.

### Colony formation assay

3,000 cells were seeded on each 10 cm culture dish. After 14 days, colonies were visualized by staining with 1% crystal violet. The number and size of colonies were counted and measured using ImageJ (NIH, Bethesda, MD), and differences were assessed by two-tailed *t*-test.

### Cell viability assay

Cells were plated and were transfected in 96-well plates. After 4 days of culture, cell viability was determined using the CellTiter-Glo Luminescent Cell Viability Assay (Promega).

### Time-dependent cell proliferation assay

To measure cell proliferation over time, cells were seeded and treated in 96-well plates. Cells were then placed in an Incucyte Zoom live cell imaging system (Essen Bioscience, Ann Arbor, MI) to monitor cell confluence every 6 hours for 9 days. The cell proliferation rates between different treatments were compared using Prism 7 (GraphPad Software, San Diego, CA).

### BrdU incorporation assay

Cell proliferation was measured using BrdU incorporation (Roche, Indianapolis, IN). Briefly, cells were incubated with 10 μM 5-bromo-2’-deoxyuridine for 4 h, and then were fixed with FixDenat solution. Anti-BrdU-POD was added to the fixed cells and substrate solution containing tetramethylbenzidine was used to detect and quantify the amount of BrdU incorporation.

### Quantitative RT-PCR (qRT-PCR)

mRNA levels of the indicated genes were measured using TaqMan^®^ gene expression Assays kit on an ABI PRISM 7900 Sequence Detection System (Life Technologies). Briefly, mRNA samples were first converted to cDNA by reverse transcription (RT). cDNAs equal to 25 ng mRNA were then used for polymerase chain reaction (PCR) in a total reaction volume of 10 μL. The fluorescence signals generated from the reactions were detected using the ABI PRISM 7900 Sequence Detection System. Three replicate PCR reactions were performed for each sample. GAPDH mRNA levels were measured as an internal control for RNA loading. Threshold cycle numbers (C_t_) were obtained and relative gene expression was calculated using the comparative cycle number method.

### Western blot

Cell lysates were prepared using RIPA buffer. Protein concentration was determined using the Pierce BCA assay (Thermo Fisher, Rockford, IL). For electrophoresis, 30 μg of protein lysate per sample were resolved by SDS-PAGE and transferred to Immun-Blot PVDF membranes (Bio-Rad, Hercules, CA). Membranes were blocked and probed with the following antibodies: mouse anti-Flag antibody (Sigma, Cat. No. F1804), mouse anti-LMO1 antibody (Sigma, Cat. No: SAB1404028), rabbit anti-TTK antibody (Cell Signaling Technology, Cat. No. 5469S), or rabbit anti-calnexin (Santa Cruz Biotechnology, Cat. No. SC-11397). Bound antibodies were detected using secondary antibodies conjugated with horseradish peroxidase (HRP) (Santa Cruz Biotechnology) and visualized by enhanced chemiluminescent (ECL) substrate (Pierce/Thermo Fisher). Band intensities were quantified using ImageJ.

### Animal experiments

Female athymic nude Foxn1nu (nu/nu) mice six to eight weeks old were purchased from Harlan-Sprague-Dawley (Indianapolis, IN). To generate tumor xenografts, 2×10^6^ H1993 cells in 200 μL PBS were injected subcutaneously into the right flanks. Tumor volumes were measured using calipers every 3 days. At the end of the study, tumor samples were isolated and snap frozen in liquid nitrogen and stored at -80°C. To measure tumor protein levels, protein lysates from each treatment group were pooled, and proteins were detected by western blot using specific antibodies. Animal protocols were approved by the Institutional Animal Care and Use Committee of the University of Texas Health Science Center at San Antonio.

### mRNA expression profiling of cell lines and tumor specimens

As described in Girard, et al. [23], total RNA was isolated using TRIzol Reagent (Life Technologies) and quantified using a Nanodrop 1000 spectrophotometer (Thermo Fisher). For tumor specimens, 10-20 μm thick serial sections of surgically resected NSCLC specimens were obtained using a Leica cryostat and homogenized using an Omni TH homogenizer (Omni International, Kennesaw, GA): total RNA was isolated using TRIzol Reagent and quantified as above. RNA quality was determined on an Agilent 2100 Bioanalyzer (Agilent Technologies, Santa Clara, CA). For mRNA profiling, total RNA samples were labeled using the mRNA Complete Labeling and Hyb Kit and hybridized to human WG-6 v3.0 Expression BeadChips (Illumina, San Diego, CA) following standard protocols. The arrays were scanned on an Agilent DNA Microarray Scanner (Agilent Technologies). mRNA expression levels were extracted using the Feature Extraction software (Agilent Technologies) and pre-processed using the R package mbcb for background correction [42]. The arrays were then log-transformed and quantile-normalized.

### Patient survival analysis

Two datasets were used in this study: the MDACC dataset was kindly provided by Dr. Ignacio Wistuba from Departments of Pathology and Thoracic/Head and Neck Medical Oncology at UT MD Anderson Cancer Center (MDACC); the Director’s Challenge Adenocarcinoma Lung Study dataset was obtained from NCI caBIG caArray (cabig.cancer.gov). To examine the association between mRNA levels and lung cancer patient survival, patients from the MDACC dataset were stratified by tumor mRNA levels. The high and low expression groups for each mRNA were defined to include patients with tumor mRNA levels ranking in the top and bottom quintiles of the population. The statistical significance of the difference in tumor mRNA levels between the two groups was assessed by Student’s *t*-test. Overall and recurrence-free survival curves were plotted using Prism 7 (GraphPad, La Jolla, CA), and statistical significance was assessed by Mantel-Cox log-rank test. Multivariate analysis was used to examine whether LMO1 mRNA level is an independent predictor of lung cancer patient survival in both the MDACC and Director’s Challenge datasets; the stratification of the variables in the analysis is described in Supplementary Table 2.

### Other statistical analyses

One-way ANOVA and Tukey’s multiple comparison tests were used to assess significance of differences in average LMO1 mRNA levels between the different histological groups of lung cell lines. For all other conditions, the statistical significance of differences between treatment and control groups was determined by *t*-test, with p< 0.05 considered statistically significant.

## SUPPLEMENTARY MATERIALS FIGURES AND TABLES


