# Supplementary material for: LMO1 functions as an oncogene by regulating TTK expression and correlates with neuroendocrine differentiation of lung cancer
Source: Oncotarget. 2018 Jul 3;9(51):29601–18. doi: 10.18632/oncotarget.25642 (PMC6049873; doi:10.18632/oncotarget.25642)
Supplement: Supplementary file 1 [file oncotarget-09-29601-s001.pdf]

LMO1 functions as an oncogene by regulating TTK expression and correlates with neuroendocrine differentiation of lung cancer

SUPPLEMENTARY MATERIALS

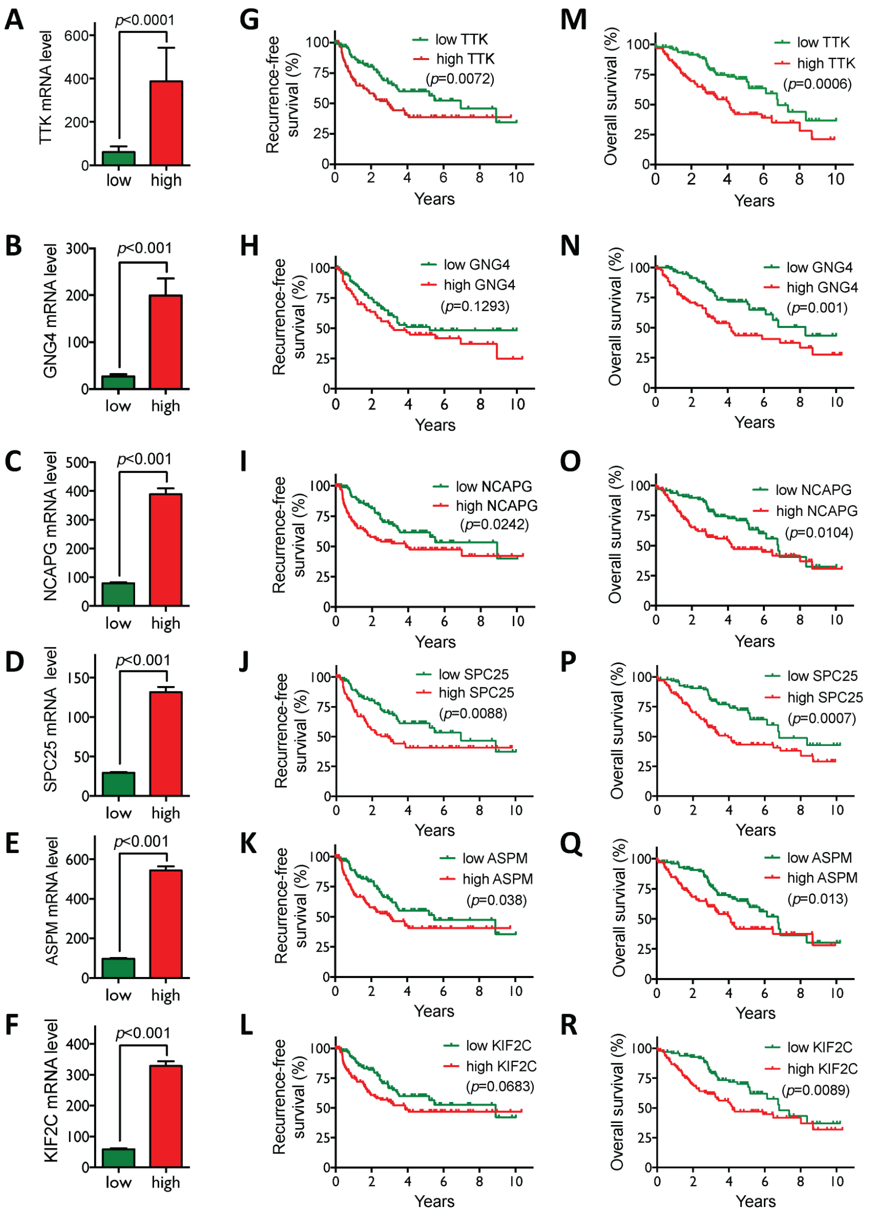

Supplementary Figure 1: Correlations of mRNA levels with lung cancer patient survival. For each of the six genes, patients with mRNA levels in the highest and lowest quintiles were selected from the 245 NSCLC patients in the MDACC dataset, defining high and low expression groups (n=50/group). Shown are differences in mRNA levels **(A-F)**, recurrence-free survival **(G-L)** and overall survival **(M-R)** for the six genes. Statistical significance of differences in mRNA levels between the two groups was assessed by Student's *t*-test. Statistical significance of differences in survival between the two groups was assessed by Mantel-Cox log-rank test.

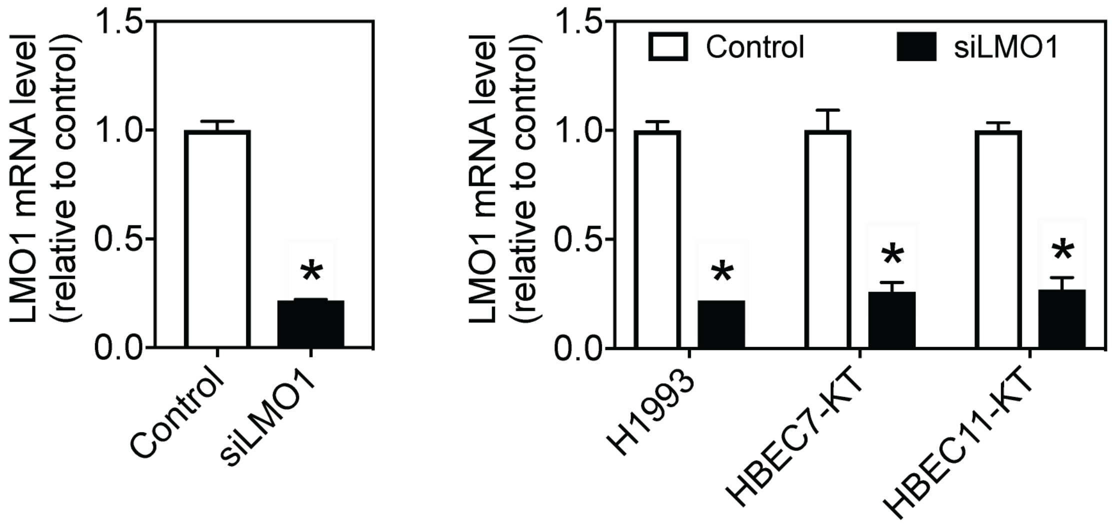

Supplementary Figure 2: Knock-down of LMO1 by siRNA in HBEC7-KT and HBEC-11KT cells. HCC827 **(A)**, H1993, HBEC7-KT and HBEC11-KT **(B)** cells were transfected with either control or siLMO1 siRNA at 25 nM. After 72 h, RNA was isolated from cells, and mRNA levels of LMO1 were measured as above. *, $p<0.05$, relative to control.

Supplementary Table 1: Genetic backgrounds of cell lines used in this study.

(a) Cell Line	(b) Tumor Subtype	(c) Age (yr)	(d) Gender	(e) TP53	(f) CDKN2A	(g) KRAS	(h) LMO1 mRNA expression	(i) Grouping	(j) TTK mRNA expression
H69	SCLC	53	M	E171*	WT	WT	1362	H	1419
H157	Squamous cell carcinoma	59	M	E298*	E69*	G12R	49	M	966
H358	Adenocarcinoma	UNK	M	HD	WT	G12V	25	L	1222
H378	SCLC	66	F	Y163C	WT	WT	738	H	1702
H460	Large cell	UNK	M	WT	del	Q61H	43	M	856
H524	SCLC	63	M	T155N	WT	WT	1396	H	863
H1155	Large cell neuroendocrine	36	M	R273H	WT	Q61H	99	H	653
H1299	Large cell neuroendocrine	43	M	HD	WT	WT	35	L	1750
H1437	Adenocarcinoma	60	M	R267P	del	WT	148	H	199
H1648	Adenocarcinoma	39	M	104_105 ins	1_457del	WT	61	M	340
H1770	Neuroendocrine	57	M	R248W	WT	WT	49	M	918
H1993	Adenocarcinoma	47	F	C242W	WT	WT	226	H	310
H2009	Adenocarcinoma	68	F	R273L	WT	G12A	42	M	1334
H2122	Adenocarcinoma	46	F	C176F, Q16L	del	G12C	47	M	514
H2126	Adenocarcinoma	65	M	E62*	1_471del	WT	29	L	728
HCC15	Squamous cell carcinoma	55	M	D259V	del	WT	258	H	1997
HCC44	Adenocarcinoma	54	F	R175L	WT	G12C	29	L	572
HCC78	Adenocarcinoma	55	M	S241F	R58X	WT	26	L	663
HCC366	Adenocarcinoma	80	F	Y220C	WT	WT	36	L	887
HCC827	Adenocarcinoma	38	F	V218-	UNK	WT	24	L	936
HBEC7-KT	Normal bronchial epithelial	66	F	N/A	N/A	N/A	24	L	86
HBEC11-KT	Normal bronchial epithelial	73	F	N/A	N/A	N/A	34	L	295

Shown are the **(a)** cell line name, **(b)** tumor subtype or tissue type from which the cell line is derived, **(c)** age and **(d)** gender of the patient, **(e-g)** identified mutations in the (e) TP53, (f) CDKN2A and (g) KRAS genes, **(h)** LMO1 mRNA level presented as signal intensity from the Illumina microarray after extracting the background signal, (i) LMO1 mRNA level grouping and **(j)** TTK mRNA expression presented as signal intensity from the Illumina microarray after extracting the background signal. UNK, unknown; F, female; M, male; HD, homozygous deletion; del, deletion; ins, insertion; WT, wildtype; N/A, not applicable. L, low LMO1 group (<40); M, medium LMO1 group (≥040 and <100); H, high LMO1 group (≥100);

Supplementary Table 2: Characteristics of patients in the MDACC and Director's Challenge datasets.

(a) Variable	(b) MD Anderson Dataset (n=245)	(c) Director's Challenge Dataset (n=440)
Ethnicity	12 African American	12 African American
	5 Asian	6 Asian
	2 Asian or Pacific Islander	1 Hawaiian or Pacific Islander
	218 Caucasian	292 Caucasian
	8 Hispanic	0 Hispanic
	0 Unknown	129 Unknown
Diagnosis	169 Adenocarcinoma	440 Adenocarcinoma
	76 Squamous Cell Carcinoma	0 Squamous Cell Carcinoma
Age	64.5 y (9.9 y)	64.4 y (10.1 y)
Gender	112 females	218 females
	133 males	222 males
Tobacco	23 never	48 never
	114 current	32 current
	106 former	267 former
	2 recently quit	93 unknown
T stage	56 T1	150 T1
	135 T2	251 T2
	14 T3	28 T3
	25 T4	11 T4
	15 unknown	0 unknown
N stage	138 N0	299 N0
	47 N1	87 N1
	45 N2	53 N2
	15 unknown	1 NX
Therapy	39 neoadjuvant, 0 unknown	88 adjuvant CT, 120 unknown
	99 adjuvant, 1 unknown	64 adjuvant RT, 121 unknown

Shown are the **(a)** variables and **(b-c)** corresponding numbers of patients in the two datasets.

Supplementary Table 3: Coding of parameters for proportional hazards regression.

Variable	Coding
LMO1	Tumor level of LMO1 mRNA
Age	Age at diagnosis in years
Gender	Female=1, Male=2
Tobacco	Current=1, Former=2, Never=3
T stage	T1=1, T2=2
N stage	N0=1, N1=2

Shown are the names of the variables and the coding of the corresponding variables.

Supplementary Table 4: Candidate genes mediating LMO1 function.

(a) Symbol	(b) Gene Name	(c) Chromosomal location	(d) Biological Process
TTK	TTK protein kinase	6q13-q21	protein phosphorylation, spindle organization, mitotic spindle organization
GNG4	Guanine nucleotide binding protein (G protein), gamma 4	1q42.3	regulation of G-protein coupled receptor signaling pathway
NCAPG	Non-SMC condensin I complex, subunit G	4p15.33	cell cycle, mitosis, mitotic chromosome condensation, cell division
ASPM	Asp (abnormal spindle) homolog, microcephaly associated (Drosophila)	1q31	cell cycle, mitosis, forebrain neuroblast division
SPC25	SPC25, NDC80 kinetochore complex component, homolog (S. cerevisiae)	2q31.1	cell cycle, mitotic spindle organization, chromosome segregation, mitosis
KIF2C	Kinesin family member 2C	1p34.1	microtubule-based movement, microtubule depolymerization, cell cycle, mitosis

Shown are the **(a)** gene symbol, **(b)** gene name, **(c)** chromosomal location, and **(d)** biological processes in which the gene is involved.

Supplementary Table 5: Student's *t*-test of the mean LMO1 mRNA levels between paired histological groups of lung cell lines.

Groups	*p* value	Significant?
Normal vs. NSCLC	0.0028	Yes
Normal vs. SCLC	<0.0001	Yes
NSCLCs vs. SCLC	<0.0001	Yes

Shown are the paired groups, *p* value and statistical significance based on the $p<0.05$ cut-off. LMO1 mRNA levels were measured as above.
